# The new nano-enabled phase map of ZrO_2_-Al_2_O_3_

**DOI:** 10.1038/s41598-019-42058-4

**Published:** 2019-04-03

**Authors:** Iwona Koltsov, Giora Kimmel, Svitlana Stelmakh, Kamil Sobczak, Witold Lojkowski

**Affiliations:** 1 Institute of High Pressure Physics, Polish Academy of Sciences, Sokolowska 29/37, 01-142 Warsaw, Poland; 2 Ben-Gurion University of the Negev, Institutes for Applied Research & Materials Engineering Department, Beer-Sheva, 84105 Israel; 3 Faculty of Chemistry, Biological and Chemical Research Centre, University of Warsaw, Żwirki i Wigury 101, 02-089 Warsaw, Poland

## Abstract

Rapid development of nanotechnology often requires verification of existing phase diagrams, which were suitable for bulk materials. This work presents a new phase map (phase diagram) for Al_2_O_3_-ZrO_2_ crystalline powders including the role of the nanoscale particles. Al_2_O_3_-ZrO_2_ composites are relevant for industry for applications demanding high hardness. The nanopowders were manufactured via co-precipitation process followed by microwave hydrothermal synthesis (MHS) at 270 °C, drying at room temperature and annealing in the temperature range 300–1500 °C. The phase composition was investigated using X-ray diffraction (XRD) and Rietveld refinement analysis. The grain size and size distribution were calculated using Rietveld refinement analysis and using transmission electron microscopy (TEM). A particular feature of the composites was the nanoisolation, separation of different phases on a nanoscale. This feature limited grain growth during annealing and permitted the phase diagram for a nano-enabled system to be determined, which turned out to be different from that of conventional composites. In particular, considerable solubility of Al^3+^ in ZrO_2_ was found for temperatures less than 1000 °C.

## Introduction

Alumina-toughened zirconia (ATZ- ZrO_2_-Al_2_O_3_) and zirconia-toughened alumina (ZTA- Al_2_O_3_-ZrO_2_) are important materials for high-temperature structural^1–3^ and functional^4–7^ applications thanks to their exceptional mechanical properties, and high temperature stability.

These ceramic materials can be synthesized using various methods. The most popular methods are: the sol–gel method^8^, hydrothermal synthesis^9^ and the co-precipitation followed by calcination^10^. The above mentioned synthesis routes yield nano- and microsized materials. Recently, we evaluated the advantages of producing ZrO_2_-Al_2_O_3_ materials using 4-step method: (I) co-precipitation of precursors, (II) microwave hydrothermal synthesis (MHS), (III) drying materials in room temperature, and (IV) annealing^11,12^. This method allows producing highly crystalline nanoparticles with a narrow particle size distribution.

Unique physico-chemical properties of nanomaterials prompted an unprecedented interest in control of their nanostructure and characterization. In most cases bulk material properties differ significantly from those obtained for materials of the same chemical composition but with grain size in the micrometer range. Kimmel *et al*.^13^, discussed the role of the grain size on the coexistence of ZrO_2_-Al_2_O_3_ phases. According to Kimmel *et al*.^13^, nanoscale grain size leads to formation of phases and structures which do not appear for ZrO_2_-Al_2_O_3_ conventional composites and/or are metastable. Materials obtained in sol-gel process by Kimmel *et al*.^13^ were identified as tetragonal zirconia (t-ZrO_2_), quasi-amorphous zirconia and several spinel-like aluminas. These findings are consistent with several earlier reports^14–17^.

In the case of micron-sized ZrO_2_-Al_2_O_3_ powders, at ambient pressure and below 1530 °C, there is no mutual solubility of the two oxides. However the situation changes when the grain size of the oxides is reduced to the nano-range^13^. In nanopowders dissolution of Al^3+^ in zirconia can take place^13,18–20^. According to Kimmel *et al*.^13^, the Zr-rich side of the binary oxides phase diagram shows quasi-amorphous ZrO_2_ and t-ZrO_2_–like structures after annealing of xerogels at low temperatures. In addition, in the region from ZrO_2_ – 0% Al_2_O_3_ up to ZrO_2_ – 25% Al_2_O_3_, temperature up to 1100 °C, and grain size below 50 nm stable t-ZrO_2_ solid solution was found. After annealing xerogels above 1200 °C, the two oxides separate and stable mixture of the monoclinic phase of ZrO_2_ (m-ZrO_2_) and corundum (α-Al_2_O_3_) was present.

Thus, phase diagrams for nanomaterials may be different from that of conventional materials. Understanding of nanomaterials phase composition is critical for many industrial applications such for example additive manufacturing or smart nanocomposites. Since the initial MHS products are different than products obtained in sol-gel method it is essential to prepare a new phase diagram of the ZrO_2_-Al_2_O_3_ system for nanosized particles obtained in MHS in a wide annealing temperature and composition range.

## Results

The present method of particles preparation ensured that the grain size of particles was in the nano range from room temperature up to 1000 °C for the identified several phases present for the composition range from 0% Al to 100% Al.

The Boehmite (AlOOH) grain size evaluated by Rietveld refinement analysis for as-synthesized samples was found to be above 100 nm. When it transforms to γ-Al_2_O_3_, grain size of that phase was 10 nm and stayed in this range up to 700 °C. At 1000 °C, for γ-Al_2_O_3,_ the grain size was found to be up to 15 nm. Upon further annealing, when the grain size increases above 15 nm, the phase transition γ- to θ-Al_2_O_3_ takes place. The grain size of t-ZrO_2_ was in the same range as alumina.

**Figure 1:**
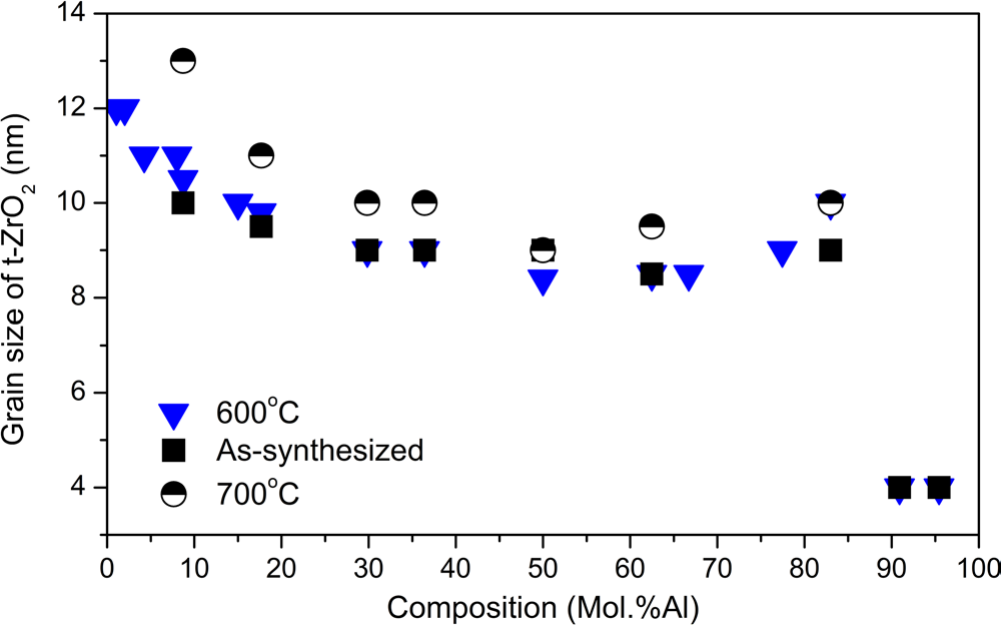
t-ZrO_2_ grain size as a function of composition.

Figure 1 presents how the grain size of tetragonal zirconia depends on its composition, for as synthesized samples, annealed at 600 °C, and at 700 °C. The t-ZrO_2_ grain size is in the range 4–12 nm for as synthesised samples and samples annealed at 600 °C. Increase of annealing temperature up to 700 °C causes minor grain growth up to 13 nm. Annealing at 1000 °C leads to the grain size increase from 20 to 60 nm only and these values were found for 85% and 10 mol.% of Al, respectively. Annealing in the temperature range 1000–1200 °C caused further increase up to 100 nm. For this phase a solid solution of Al^3+^ in ZrO_2_ was observed for Al content up to 30 mol% and temperature below 1000 °C. In contrast to the sol-gel products (as-synthesised and after annealing) there was no zirconia-like quasi amorphous phases in any powder obtained in MHS^12,13^.

**Figure 2:**
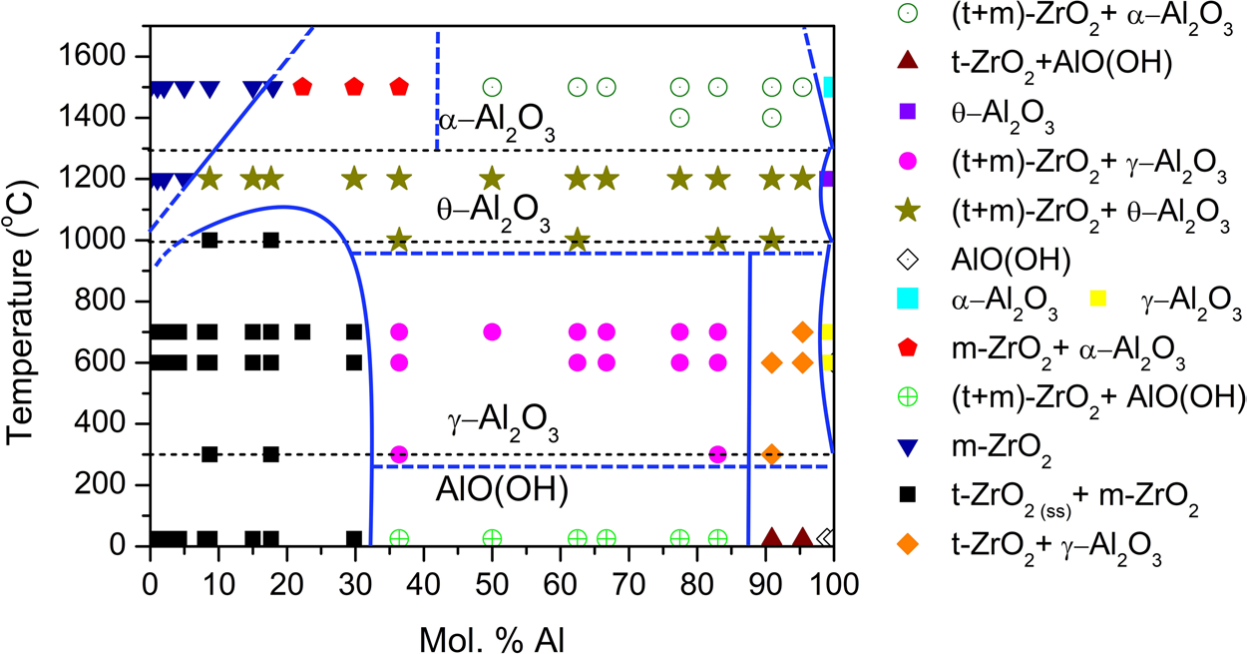
Nano-enabled phase composition map for the ZrO_2_-Al_2_O_3_ system.

Figure 2 shows the new phase map for ZrO_2_-Al_2_O_3_ nanopowders. The phase map was determined for as-synthesized ZrO_2_-Al_2_O_3_ nanomaterials and powders after annealing for 1 h in whole range of compositions in temperatures range from 300 to 1500 °C. Samples with 1 wt.% or less of a given phase were considered free of that phase. The symbols visible in Fig. 2. represent phase mixtures (i.e. a star: mixture of t-ZrO_2_, m-ZrO_2_, θ-Al_2_O_3_), as well as single phases (i.e. a square: α-Al_2_O_3_). Dotted black lines marked in Fig. 2. represent the temperatures of alumina phase transitions and they were determined using thermal analysis^21^ and Rietveld refinement.

**Figure 3:**
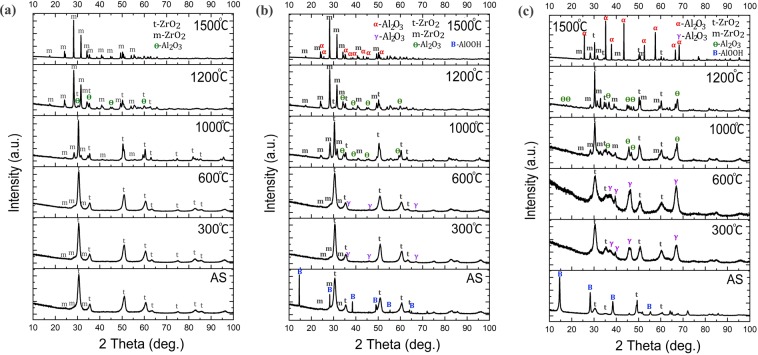
XRD patterns collected from room temperature up to 1500 ^o^C for chosen compositions, where: (**a**) is 19 mol. % of Al, (**b**) is 36 mol.% of Al, and (**c**) represents 94 mol.% of Al.

For better clarity and understanding of presented phase map (Fig. 2) the XRD patterns for chosen compositions are presented in Fig. 3. All existing phases were marked in the figure. It should be noted, that phase map was determinate for crystalline phases. During phase analysis of compositions with high amount of Al we found that after Boehmite (AlOOH) disappearance and from ~700 °C weak, very broad, γ-Al_2_O_3_ diffraction lines appear with very high background (Fig. 3c). That may indicate presence of residual amorphous alumina phase.

**Figure 4:**
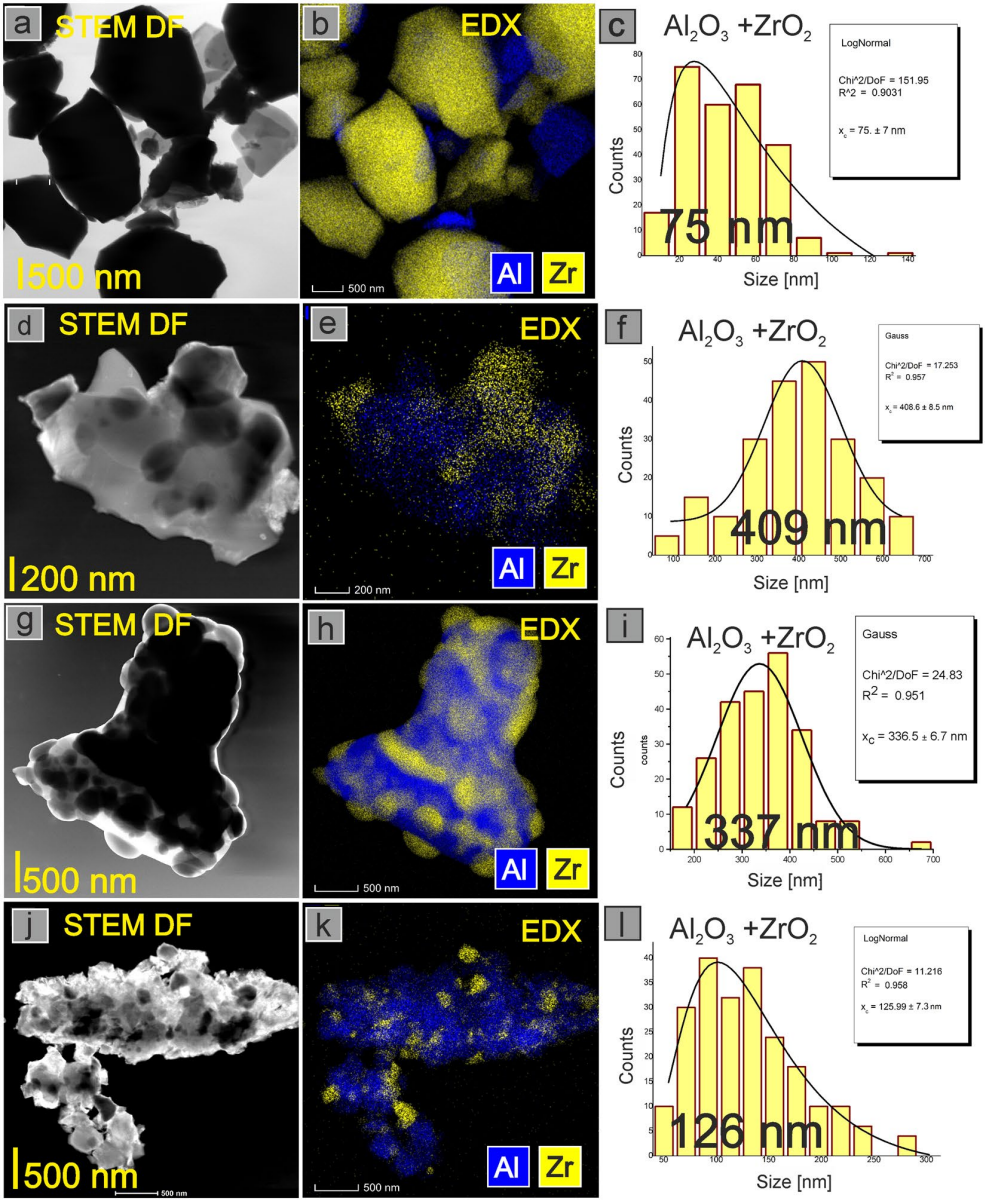
TEM pictures, EDX, and particle size distribution of ZrO_2_ and Al_2_O_3_ for the compositions with following Al amount: 10 mol.% (**a**–**c**), 50 mol.% (**d**–**f**), 70 mol.% (**g**–**i**), 90 mol.% (**j**–**l**) of Al, after annealing at 1500 ^o^C.

Since this work is dedicated to size dependent phase map of ZrO_2_-Al_2_O_3_ nanopowders we applied 2 methods to evaluate grain size. For grain sizes above 60 nm Rietveld analysis cannot be applied and TEM investigations were needed to determine particle size distribution for samples annealed at 1500 °C (Fig. 4). The morphology of ZrO_2_ and Al_2_O_3_ nanopowders obtained using the 4-steps method was discussed previously^11,12^. The first column of images in Fig. 4 shows the change of morphology, the second column shows the elements distribution (EDX), and the third one shows particle size distribution for ZrO_2_ and Al_2_O_3_ particles. The compositions chosen for TEM study were as follows: ZrO_2_ with 10 mol.% of Al (Fig. 4a–c), ZrO_2_ with 50 mol.% of Al (Fig. 4d–f), ZrO_2_ with 70 mol.% of Al (Fig. 4g–i), and ZrO_2_ with 90 mol.% of Al (Fig. 4j–l). Figure 4 shows that the nano-particles of the component phases formed agglomerates. The agglomerates size depends on composition. It is seen that the individual particles size of ZrO_2_ is in the range from 50 up to 500 nm for powders containing 90 and 10 mol.% of Al, respectively. Thus, in the whole composition range, up to 1500 °C, the particle size of at least one of the components is in the nano-range. For the annealing temperature lower than 1000 °C all the system components displayed size less than 60 nm. Hence, the phases map studied in the present paper concern a nano-enabled system.

**Figure 5:**
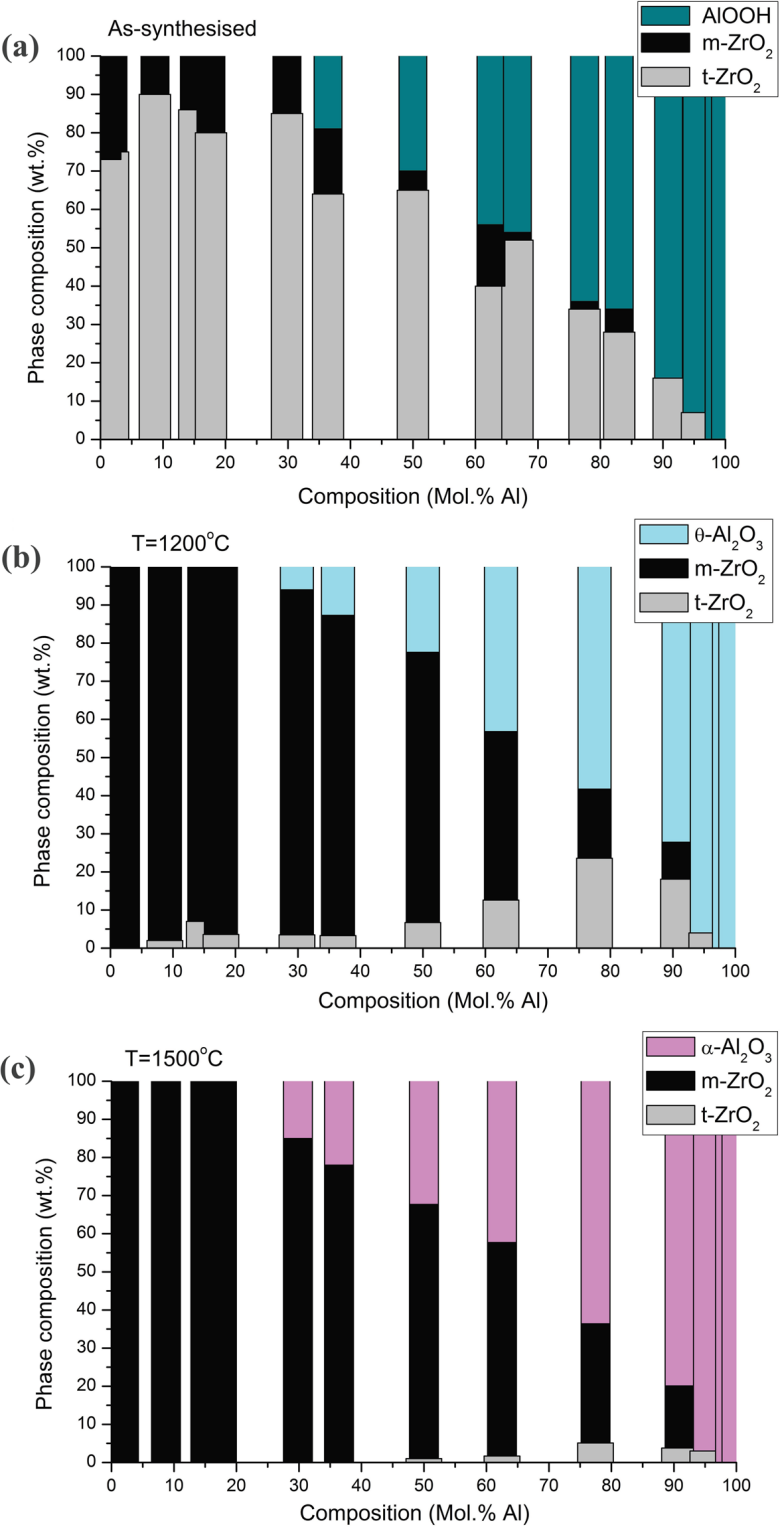
Relation between wt.% amount of t-ZrO_2_, m-ZrO_2_ and Al-phases as a function of composition and annealing temperature.

Figure 5a–c presents the change of content in the system of t-ZrO_2_, m-ZrO_2_, AlOOH, θ-Al_2_O_3_ and α-Al_2_O_3_ phases as a function of composition and annealing temperature. Figure 5a presents results for as synthesized materials, while Fig. 5b,c show change of composition for powders after annealing at 1200 °C, and 1500 °C, respectively. The graphs were obtained based on Rietveld refinement analysis performed for XRD patterns.

## Discussion

It is well known, that zirconia exists in three polymorphic phases: monoclinic (m), stable up to 1170 °C, tetragonal (t), stable up to 2370 °C and cubic (c), stable up to 2715 °C^12^. Usually, a stable at room temperature t-ZrO_2_ phase is obtained by doping zirconia with some elements i.e. Mg, Ca, Re (Y etc.). Such stabilization is achieved by incorporation of divalent or trivalent cations in the fluorite-type structure by substitution of Zr^4+^ cations and creation of oxygen vacancies to maintain the local charge balance^22^. Stabilized zirconia can be processed in a wide temperature range without phase transformations, which is especially important for sintering.

Srdic *et al*.^22^, discussed stabilization of t-ZrO_2_ using Al^3+^ ions. Authors noticed that development of nanoprocessing techniques in which precursors are mixed on the molecular level have enabled the formation structures, where Al^3+^ is incorporated into the zirconium-oxygen network^12,13,22^. One of methods that allows to stabilize t-ZrO_2_ at room temperature and forms a t-ZrO_2_ solid solution (t-ZrO_2(ss)_) is co-precipitation of ZrO_2_ and Al_2_O_3_ precursors together with microwave hydrothermal synthesis, which was reported before^11,12^. It is frequently claimed^22^ that doping with ions of lower valency than zirconia leads to vacancy formation to ensure charge compensation, and stabilization of the t-ZrO_2_ structure. In our opinion for the nano-sized particles’ stabilization is caused not by doping but the small size and surface energy contribution, and this is in agreement with^23–25^. Further, charge compensation effects are hardly imaginable for such high doping levels. It is highly improbable that each Al^3+^ is accompanied by a vacancy for example 20 or 30 mol. % addition, as there cannot be 20–30% of sites occupied by vacancies. Thus, at nanoscale it is possible to form a regular solid solution of Al^3+^ in zirconia due to stability of the t-ZrO_2_ phase caused by the very small grain size.

Discussion on the effect of composition on agglomerates size is outside the scope of the present paper. Here we are concerned only with t-ZrO_2_ grain size influence. Remarkable is that the aluminum rich and zirconia rich particles are homogeneously dispersed. As discussed in^11^ this leads to a nanoisolation effect, resulting from the 4-steps synthesis procedure. Nanoisolation means that nanoparticles of zirconia or zirconia rich phases are separated from each other by aluminum-rich phases, and vice-versa. Nanoisolation hinders particle growth due to Oswald ripening and coalescence. It is also a plausible reason for limited particles size growth observed in the present paper, which permitted to construct the nano-enabled phase diagram.

As it can be seen from Fig. 1 the grain size of t-ZrO_2_ decreases with increasing mol.% of Al. The smallest t-ZrO_2_ grain size was found for ZrO_2_ with 90 mol.% and more of Al. The grain of size of t-ZrO_2_ did not change meaningfully during annealing powders up to 700 °C but increases slightly after annealing at 1000 °C. The biggest t-ZrO_2_ grain size after treatment at 1000 °C was found for the lowest Al content. For the small particles below 15 nm, Al^3+^ dissolve in the crystal structure of ZrO_2_, and segregation of alumina does not take place up to 1000 °C. This is especially visible at high temperatures were isolated in alumina matrix nanosized t-ZrO_2_ grains do not undergo transformation into m-ZrO_2_.

Figure 2 presents the phase map for ZrO_2_-Al_2_O_3_ nanocrystalline powders. It can be observed, that t-ZrO_2_-based solid solution, coexisting with a small amount of m-ZrO_2_ (up to 20 wt.%) is present up to 30 mol. % of Al, in the annealing temperature range up to 1000 °C. The solid solution formula is Zr_(1−x)_Al_x_O_(2−x/2)_, where x~0.45. The phase transformation of solid solution during temperature increase for compositions up to 30 mol.% of Al is presented below (1):

1 $${{\rm{t}} \mbox{-} \mathrm{ZrO}}_{2({\rm{ss}})}\,+\,{{\rm{m}} \mbox{-} \mathrm{ZrO}}_{2}\mathop{\longrightarrow }\limits^{\sim 1000\,^\circ C}{t \mbox{-} \mathrm{ZrO}}_{2}\,+\,{{\rm{m}} \mbox{-} \mathrm{ZrO}}_{2}\,+\,{{\rm{\theta }} \mbox{-} \mathrm{Al}}_{{\rm{2}}}{{\rm{O}}}_{{\rm{3}}}\mathop{\longrightarrow }\limits^{\sim 1200\,^\circ C}{{\rm{m}} \mbox{-} \mathrm{ZrO}}_{2}\,+\,{{\rm{\alpha }} \mbox{-} \mathrm{Al}}_{{\rm{2}}}{{\rm{O}}}_{{\rm{3}}},$$

where t-ZrO_2(ss)_ is the solid solution of Al^3+^ in t-ZrO_2_. Above 1000 °C the grains start to grow, and at the same time θ-Al_2_O_3_ precipitates. At 1200 °C all components of ZrO_2_-Al_2_O_3_ system fully precipitate. These findings are in agreement with our previous work^21^. Thermal treatment longer than 1 h in the temperature range from 1100 °C up to 1500 °C lead to gradual phases segregation according to the phase diagram for conventional materials. The change of phase composition for this region is in addition presented in Fig. 3(a). It can be seen that for all compositions below 19% of Al after annealing at 1500 °C only m-ZrO_2_ phase exists.

From 36% up to 84 mol.% of Al in as-synthesized samples three phases were present: tetragonal, monoclinic zirconia and the crystalline Boehmite. Boehmite undergoes further transformation to γ-Al_2_O_3_ at approximately 300 °C (Fig. 2). These findings are in agreement with our previous studies^12^. Samples with compositions in the range from 36 to 84 mol. % of Al follow phase transitions (2) as reported in literature^11,17,25–27^.

2 $$\gamma {\textstyle \text{-}}{\rm{A}}{\rm{l}}{\rm{O}}({\rm{O}}{\rm{H}})\,({\rm{B}}{\rm{o}}{\rm{e}}{\rm{h}}{\rm{m}}{\rm{i}}{\rm{t}}{\rm{e}})\,\mathop{\longrightarrow }\limits^{300{\textstyle \text{-}}{600}^{\circ }C}\,{\gamma {\textstyle \text{-}}{\rm{A}}{\rm{l}}}_{2}{{\rm{O}}}_{3}\,\mathop{\longrightarrow }\limits^{\sim {1000}^{\circ }C}\,{\theta {\textstyle \text{-}}{\rm{A}}{\rm{l}}}_{2}{{\rm{O}}}_{3}\,\mathop{\longrightarrow }\limits^{1200{\textstyle \text{-}}{1250}^{\circ }C}\,{\alpha {\textstyle \text{-}}{\rm{A}}{\rm{l}}}_{2}{{\rm{O}}}_{3}$$

In addition, Fig. 3(b) shows change of XRD patterns for 36 mol. % of Al as a function of temperature.

Above 36 mol.% of Al at temperatures above 1200 °C segregation of Al_2_O_3_ is fast and it is complete at 1500 °C. The crystalline phase transformation of ZrO_2_-Al_2_O_3_ in referred composition range is shown below (3):

3 $$\begin{array}{c}\mathrm{AlO}(\mathrm{OH})\,\mathop{\longrightarrow }\limits^{300\mbox{--}600\,^\circ C}\,{t \mbox{-} \mathrm{ZrO}}_{2}\,+\,{{\rm{m}} \mbox{-} \mathrm{ZrO}}_{2}\,+\,{{\rm{\gamma }} \mbox{-} \mathrm{Al}}_{2}{{\rm{O}}}_{3}\,\mathop{\longrightarrow }\limits^{\sim 1000\,^\circ C}\,{{\rm{t}} \mbox{-} \mathrm{ZrO}}_{2}\,+\,{{\rm{m}} \mbox{-} \mathrm{ZrO}}_{2}\\ \,+\,{{\rm{\theta }} \mbox{-} \mathrm{Al}}_{2}{{\rm{O}}}_{3}\,\mathop{\longrightarrow }\limits^{\sim 1200\,^\circ C}\,{\rm{t}} \mbox{-} \mathrm{ZrO2}\,+\,{\rm{m}} \mbox{-} \mathrm{ZrO2}\,+\,{{\rm{\alpha }} \mbox{-} \mathrm{Al}}_{2}{{\rm{O}}}_{3}\end{array}$$

In the last region of ZrO_2_-Al_2_O_3_ phase map from 90 to 100% of Al (Fig. 2) m-ZrO_2_ phase disappears in as-synthesized nanopowders from 90 up to 94 mol.% of Al. From 98 mol.% to 100% of Al pure AlO(OH) phase exists. All Al-based phases transform into alumina transition phases in the way described by Levin *et al*.^17^. The change of phases with temperature for 94 mol.% of Al is shown in Fig. 3(c).

Inspection of Fig. 2 shows some remarkable features of the nano-enabled phase map.

- A solid solution of Al^3+^ in t-ZrO_2_ was observed for up to 30 mol.% of Al, and annealing temperature up to 1000 °C.
- Up to 96 mol.% of Al t-ZrO_2_ and m-ZrO_2_ coexisted, with m-ZrO_2_ less than 20 wt.%, above 96 mol.% of Al, ZrO_2_ only in the tetragonal structure was found existing from RT up to 1000 °C.
- At 1000 °C precipitation of θ-Al_2_O_3_ started from the above solid solution.
- Stable γ-Al_2_O_3_ phase temperature range was from 300 to 1000 °C.
- Stable θ-Al_2_O_3_ phase temperature range was from 1000 to 1200 °C.

The above nano-enabled phase map is strikingly different from the conventional ZrO_2_ - Al_2_O_3_ phase diagram, where there is no solid solution, the t-ZrO_2_ phase is not observed below 1170 °C, and the transitions in the Al –structures takes place at temperatures ~100 °C higher than for the bulk system.

Several papers report existence of Zr_(1−x)_Al_x_O_(2−x/2)_ solid solutions for nano-sized particles^23–25^. Stefanic and Music^28^ studied crystallization of amorphous Zr_(1−x)_Al_x_O_(2−x/2)_ solid solutions obtained by precipitation. Authors^28^ showed that metastable t-ZrO_2_ solid solution crystallizes at 550–1000 °C (4), but not at lower temperature like in our case (270 °C at MHS).

4 $${\rm{Amorphous}}\,\mathop{\longrightarrow }\limits^{{\boldsymbol{\sim }}{\bf{870}}\,{\boldsymbol{^\circ }}{\boldsymbol{C}}}\,{{\rm{t}} \mbox{-} \mathrm{ZrO}}_{2({\rm{ss}})}\,\mathop{\longrightarrow }\limits^{{\boldsymbol{\sim }}{\bf{1000}}\,{\boldsymbol{^\circ }}{\boldsymbol{C}}}\,{{\rm{t}} \mbox{-} \mathrm{ZrO}}_{2}\,+\,{{\rm{\gamma }} \mbox{-} \mathrm{Al}}_{2}{{\rm{O}}}_{3}\,\mathop{\longrightarrow }\limits^{{\boldsymbol{\sim }}{\bf{1400}}\,{\boldsymbol{^\circ }}{\boldsymbol{C}}}\,{{\rm{m}} \mbox{-} \mathrm{ZrO}}_{2}\,+\,{{\rm{\alpha }} \mbox{-} \mathrm{Al}}_{2}{{\rm{O}}}_{3}$$

Contrary to our results Stefanic and Music^28^ showed, that for x > 0.3, the monoclinic phase does not appear at 1000–1100 °C. Similar to our findings Kimmel *et al*.^13^ identified solid solution in the same composition range. However, the obtained ZrO_2_-type nanopowders were quasi-amorphous after drying at room temperature. Our results showed that for compositions with higher Al amount and after thermal treatment up to 600 °C there is a significant amount of amorphous alumina (which also causes problems for the Rietveld analysis). In the phase map (Fig. 2) we refer only to the crystalline phases.

TEM method was used to evaluate particle with sizes above 60 nm, which we expected to obtain for materials after annealing at 1500 °C. The measurements were performed in STEM mode. Figure 4 shows the particle size distributions for ZrO_2_ and Al_2_O_3_ for chosen compositions after annealing at 1500 °C. The smallest particles were observed for ZrO_2_ with 10 mol.%Al (Fig. 4c), where average size was ~75 nm. Most of them are ZrO_2_ particles as confirmed by EDX studies (Fig. 4b). The average ZrO_2_ and Al_2_O_3_ particles size distribution for the composition containing higher amount of Al (50% and 70 mol.% of Al) were found to be 409 nm (Fig. 4f), and 337 nm (Fig. 4i). The nanocomposite ZrO_2_ with 90 mol.% of Al is characterized by 126 nm average ZrO_2_ and Al_2_O_3_ particle size distribution (Fig. 4l). For all compositions above 60 mol.% of Al, and after annealing at 1500 °C ZrO_2_ particles stay in nano-range in alumina matrix, what we discussed previously^11^ (Fig. 4). This finding confirms our results described above.

Figure 5 shows the percentage weight proportion of t-ZrO_2_, m-ZrO_2_ and AlO(OH) (Al_2_O_3_ at higher temperatures) phases in ZrO_2_-Al_2_O_3_ system as a function of composition and annealing temperature. These results were the crucial step for phase map construction. It can be seen from Fig. 5 that the amount of t-ZrO_2_ varies when the temperature and composition change. For ZrO_2_ up to 30 mol. % of Al and for materials in as-synthesized state the wt.% of t-ZrO_2_ is the highest and in a range from 70 to 90 wt.% (Fig. 5a). For compositions above 30 mol. % of Al the amount of t-ZrO_2_ for as-synthesized nanomaterials drops with increasing Al content. The change of ZrO_2_ wt.% vary after annealing at 1200 °C (Fig. 5b), where the segregation of Al_2_O_3_ already took place. The amount of t-ZrO_2_ drops to few percent’s in comparison to as-synthesized materials but is rising with increasing content of Al. Annealing at 1500 °C causes further lowering of t-ZrO_2_ amount (Fig. 5c), and m-ZrO_2_ becomes ZrO_2_-predominant phase. Based on the obtained results it is possible to distinguish two cases:

1. In as-synthesized materials, the t-ZrO_2_ amount decreases and m-ZrO_2_ increases with increased amount of Al.
2. Above 1200 °C the t-ZrO_2_ amount increases with increased amount of Al.

The presented above discussion was referring to the ZrO_2_-Al_2_O_3_ system phase composition for amorphous and nanocrystalline materials. The conventional phase diagram for ZrO_2_-Al_2_O_3_ bulk materials is much simpler than for the nano-enabled system or amorphous materials. Nano-enabled^29^ phase map (Fig. 2) might be useful for development of commercially viable ZrO_2_-Al_2_O_3_ composites via sintering of nanoparticles.

## Conclusions

In this work for the first time a comprehensive study of the ZrO_2_-Al_2_O_3_ system composed of nanoparticles in the wide temperature region and whole composition range was performed and the new nano-enabled phase map for the ZrO_2_-Al_2_O_3_ powders was determined. The map is considerably different comparing to the conventional phase diagram. The main difference is the presence of t-ZrO_2_ or solid solution coexisting with some m-ZrO_2_ in the range from room temperature up to ~1000 °C, while for conventional phase diagram m-ZrO_2_ phase and alumina phases only exist.

A significant role for keeping the particles size in the nano-range for the whole annealing temperatures range played nanoisolation.

We found that t-ZrO_2_ grain size before high temperature treatment is in the range from 4 to 13 nm. The smallest t-ZrO_2_ grains were found for ZrO_2_ with 90 and 94 mol% of Al. After annealing at 1500 °C the size of ZrO_2_ particles increases, but still remains in nanorange for compositions with high Al content due to zirconia nanoparticles isolation in alumina matrix.

We showed that the weight % amount of t-ZrO_2_ changes with the temperature and composition. Two trends where found. For as synthesised materials the t-ZrO_2_ amount declines with increased mol.% of Al. For materials annealed above 1200 °C t-ZrO_2_ content rises with increased amount of Al.

Presented in this work the nano-enabled phase map for ZrO_2_-Al_2_O_3_ composites is different than for bulk materials and it opens new possibilities for materials processing leading to applications.

## Materials and Methods

The procedure for obtaining of as-synthesized ZrO_2_-Al_2_O_3_ nanopowders was described in details elsewhere^21,30^. The metal oxides precursors used in the process were zirconyl chloride octahydrate (ZrOCl_2_·8H_2_O, Sigma-Aldrich (≥99.5%)), and aluminum nitrate nonahydrate (Al(NO_3_)_3_·9H_2_O, CHEMPUR, analytically pure). Microwave reactions took place in a MAGNUM II ERTEC microwave reactor (2.45 GHz, 600 W)^30^. The procedure of materials thermal treatment was described in details in our previous work^11^. The annealing process was performed for 10 min, 1 h, 2 h and in some cases more than 10 h. We found that 1 h of annealing allows alumina to segregate from the solid solution in the proper temperature range, and this annealing time was used for the phase map construction.

X-ray diffraction patterns for all ZrO_2_-Al_2_O_3_ compositions were collected using diffractometer (X’Pert PRO, PANalytical) equipped with a copper anode (CuKα1) and an ultra-fast PIXcel1D detector. The analysis took place at room temperature in the 2θ range of 10–100° with a step size of 0.03°. The obtained diffraction data were analyzed using processing methods, such as: Rietveld refinement method^31^, FullProf software^32^, and by the DBWS program^33^.

TEM investigations were conducted using an FEI Talos 20–200 transmission microscope at 200 kV, The measurements were performed in TEM mode and also in STEM using dark field (DF) imaging. Energy-dispersive x-ray spectroscopy was used to detect differences in local chemical composition. The specimens for the TEM observations were prepared by dropping the ethanol particle dispersion, created by an ultrasonic technique, on a carbon film supported on a 300-mesh copper grid. TEM tests were used to determine the nanoparticle size distribution. The grain size histograms were obtained by considering a region of a sample having about 250 nanocrystals and approximating the shape of each nanocrystal by a sphere. The obtained histograms were fitted to normal or lognormal distributions.

## Data Availability

The datasets generated during the current study are available from the corresponding author on reasonable request.

## References

[1] Chandradass J, Kim MH, Bae DS (2009). Influence of citric acid to aluminium nitrate molar ratio on the combustion synthesis of alumina–zirconia nanopowders. J. Alloy. Compd..

[2] Wei Z (2008). Preparation and property investigation of CeO2–ZrO2–Al2O3 oxygen-storage compounds. J. Alloy. Compd..

[3] Wang J, Taleff EM, Kovar D (2003). High-temperature deformation of Al2O3/Y-TZP particulate composites. Acta Mater..

[4] Yin W, Meng B, Meng X, Tan X (2009). Highly asymmetric YSZ hollow fibre membranes. J. Alloy. Compd..

[5] Benzaid R, Chevalier J, Saadaoui M (2008). Fracture toughness, strength and slow crack growth in a ceria stabilized zirconia–alumina nanocomposite for medical applications. Biomaterials..

[6] Chevalier J, De-Aza AH, Fantozzi G, Schehl M, Torrecillas R (2000). Extending the life time of ceramic orthopaedic implants. Adv. Mater..

[7] Naglieri V (2013). Optimized Slurries for Spray Drying: Different Approaches to Obtain Homogeneous and Deformable Alumina-Zirconia Granules. Materials..

[8] Shukla S, Seal S, Vij R, Bandyopadhyay S (2002). Effect of HPC and water concentration on the evolution of size, aggregation and crystallization of sol–gel nanozirconia. J. Nanopart. Res..

[9] Begand S, Oberbach T, Glien W (2007). Corrosion behaviour of ATZ and ZTA ceramic. Bioceramics..

[10] Chuang C-C, Hsiang H-I, Hwang JS, Wang TS (2009). Synthesis and characterization of Al2O3-Ce0.5Zr0.5O2 powders prepared by chemical coprecipitation method. J. Alloy. Compd..

[11] Koltsov I (2018). Mechanism of Reduced Sintering Temperature of Al2O3–ZrO2 Nanocomposites Obtained by Microwave Hydrothermal Synthesis. Materials..

[12] Malka IE, Danelska A, Kimmel G (2016). The Influence of Al2O3 Content on ZrO2-Al2O3 Nanocomposite Formation—The Comparison between Sol-Gel and Microwave Hydrothermal. Methods. Mater. Today Proc..

[13] Kimmel G (2011). XRPD study of phase transformations accompanied with grain growth in the alumina-zirconia system. Z. Kristallogr. Proc..

[14] Sato T, Osawa F, Nakamura T, Watenable H, Ikoma S (1979). Thermal decomposition of zirconium hydroxide. Thermochim. Acta.

[15] Gau G-Y, Chen Y-L (2005). A nearly pure monoclinic nanocrystalline zirconia. J. Solid State Chem..

[16] Zhou R-S, Snyder RL (1991). Structures and transformation mechanisms of the η, γ and θ transition aluminas. Acta Cryst. B.

[17] Levin I, Brandon D (1998). Metastable Alumina Polymorphs: Crystal Structures and Transition Sequences. J. Am. Ceram. Soc..

[18] Tsunekawa S, Ito S, Kawazoe Y, Wang J-T (2003). Critical Size of the Phase Transition from Cubic to Tetragonal in Pure Zirconia Nanoparticles. Nano Lett..

[19] Kikkawa S, Kijima A, Hirota K, Yamaguchi O (2002). Soft solution preparation methods in a ZrO2–Al2O3 binary system. Solid State Ionics.

[20] Li G, Li W, Zhang M, Tao K (2004). Characterization and catalytic application of homogeneous nano-composite oxides ZrO2–Al2O3. Catalysis Today.

[21] Koltsov, I., *et al*. Thermal and physical properties of ZrO2–AlO(OH) nanopowders synthesised by microwave hydrothermal method. *J Therm Anal Calorim*. **131****(**3**)**, 10.1007/s10973-017-6780-8 (2017).

[22] Srdic VV, Winterer M (2003). Aluminum-Doped Zirconia Nanopowders: Chemical Vapor Synthesis and Structural Analysis by Rietveld Refinement of X-ray Diffraction Data. Chem. Mater..

[23] Srivistawa, A. K. Oxide Nanostructures: Growth, Microstructures, and Properties, April 9, 2014 by Pan Stanford, CRC Press, ISBN 9789814411356 - CAT# N10742.

[24] Yamaguchi O, Shirai M, Yoshinaka M (1988). Formation and Transformation of Cubic ZrO2 Solid Solutions in the System ZrO2—Al2O3. J. Am. Ceram. Soci..

[25] Inamura H, Miyamoto Y, Imaida M, Takagawa K, Hirota O (1994). Formation and hot isostatic pressing of ZrO2 solid solution in the system ZrO2-Al2O3. J. Mater. Sci..

[26] Souza Santos P, Souza Santos H, Toledo SP (2000). Standard Transition Aluminas. Electron Microscopy Studies. Mater. Res..

[27] Navrotsky, A. & Ushakov, S. V. Thermodynamics of Oxide Systems Relevant to Alternative Gate Dielectrics, A. A. Demkov & A. Navrotsky (eds), Materials Fundamentals of Gate Dielectrics, Springer. Printed in the Netherlands, 10.1007/1-4020-3078-9_3, 57–108. (2005).

[28] Stefanic G, Music S (2002). Factors influencing the stability of low temperature tetragonal ZrO2. Croatica Chem. Acta..

[29] ISO/TS 80004-1:2015, Nanotechnologies — Vocabulary — Part 1: Core terms. Geneva, Switzerland, 2015.

[30] Wojnarowicz, J. *et al*. Effect of Water Content in Ethylene Glycol Solvent on the Size of ZnO Nanoparticles Prepared Using Microwave Solvothermal Synthesis. *J*. *Nanomat*. **2016**, ID 2789871, 15, 10.1155/2016/2789871 (2016)

[31] Rietveld HM (1967). A profile refinement method for nuclear and magnetic structures. J. Appl. Crystallogr..

[32] J. Rodrigez-Carvajal: Fullprof, Program for Rietveld refinement, Laboratories Leon Brillouin (CEA-CNRS), Saclay, France, 1997

[33] Young RA, Sakthivel A, Moss TS, Paiva-Santos CO (1995). DBWS-9411—An upgrade of the DBWS*.* programs for Rietveld refinement with PC and mainframe computers. J. Appl. Crystallogr..

